# Intensified Religious Pluralism and De-differentiation: the British Example

**DOI:** 10.1007/s12115-015-9984-1

**Published:** 2016-01-15

**Authors:** Linda Woodhead

**Affiliations:** Department of Politics, Philosophy and Religion, Lancaster University, County South, Lancaster, LA1 4YQ UK

**Keywords:** Religion, Religious, Spirituality, “no religion”, “nones”, Secular, Pluralism, De-differentiation, Liberal values

## Abstract

Drawing on surveys of religion and values in Great Britain, this paper suggests that Peter Berger’s paradigm of two pluralisms can be usefully supplemented by taking account of a third kind of intensified pluralism. This involves the breakdown of the boundaries between religions, and between the religious and the secular, and is therefore a pluralism of de-differentiation. It helps explain many features of contemporary religion and identity, including the rise of the “nones” and the increasing reluctance of each new generation to identify with religious (and secular) labels and packages.

Peter Berger’s two pluralisms offer us a useful new paradigm for understanding religion in the modern world. First, there is religious pluralism; second, the pluralism of religious and secular coexistence. Because these two kinds of diversity now exist together in most societies, criss-crossing one another, they result in an overall intensification of pluralism, not least in the individual mind (Berger 2014).

Here I want to suggest that this paradigm can be extended and made even more powerful by paying attention to a third kind of pluralism which has close connections with the first two, but goes beyond them. This is a pluralism of religious de-differentiation, closely tied up with a wider de-differentiation of previously more separate social domains, which many countries including Britain and the United States are experiencing today, and which in religious terms is signalized by the growing number of those reporting “no religion” – the so-called “nones”.

I came to realise the value of such an extension of the Berger paradigm in trying to make sense of several extensive surveys of the values and beliefs of the British which I carried out with the survey company YouGov in 2013 and 2014,^1^ and I will focus on the example of Britain (Scotland, England and Wales, but not Northern Ireland).

Britain is, as Berger would predict, plural in cultural and religious terms – even more so than the USA – and it also has a pluralism of the religious and the secular, de facto if not *de jure.* This is clear. But my surveys reveal an even deeper kind of pluralism, since a growing proportion of British people now refuse to categorise themselves as either religious or secular, and display notably variegated beliefs which are impossible to fit into neat religious or secular schemas. Combining this evidence with recent qualitiative studies makes it clear that the situation cannot wholly be accounted for by the fact that people are not neccesarily neatly religious in one compartment of social life (e.g., at home) and secular in another (e.g., at work). Nor do more than a tiny number identify with more than one religious tradition (like Christian and Hindu, or Quaker and Catholic). They are plural in a different sense.

## Catholics

Let me take Roman Catholics in Britain as an example, because they are in many ways the *least* likely pluralists. Not only does their tradition offer clear, centralised moral and dogmatic instruction, but they are a religious minority in Britain, with a long history of sporadic persecution and disadvantage, especially relative to the majority church, the Church of England. As such, they can be expected to have a relatively strong and rather homogenous identity, built around what Steve Bruce calls “cultural defence”.

Self-identified Catholics today constitute about 10 % of the population of Great Britain.^2^ The largest Christian denomination, Anglican, currently represents around 30 % of the population, but is declining fast. Although the proportion of Catholics is more stable, partly because of immigration, their profile is nevertheless very unlike that which their Church leaders, or the traditional categories of religious scholarship religion, would expect. Indeed, if we measure Catholics by their conformity to some of the key beliefs and behaviours laid down by the Catholic magisterium -- weekly churchgoing, certain belief in God, looking to ecclesiastical and scriptural sources for authority, opposition to abortion, same-sex marriage and euthanasia – only 5 % of Catholics qualify as “faithful” or orthodox, and a mere 2 % of Catholics aged under 30 (in Britain at least it is a myth that younger Catholics are on the whole more orthodox than their elders).

Nor do a majority of Catholics label themselves “religious.” Only a fifth describe themselves as such, a tenth as “spiritual,” and another fifth as “both.” The greatest proportion, one third, say “I would not describe myself, or my values and beliefs, as spiritual or religious.” Overall, a quarter of self-identified Catholics do not believe in God, and over half do not go to church. A majority do not identify as either “religious” or “spiritual”, when presented with a range of other options. A third say that they practice no spiritual activity in private. And, rather delightfully, one in five self-identified Catholics report that they are “not influenced by any religion at all”.

The official Catholic Church would, and regularly does, say that this shows that Catholics need better instruction and discipline. Older paradigms in the study of religion say that they are gradually secularising. Both are asserting that Catholics have departed from the norm of what these authorities consider to be “real religion”, but my surveys show that neither offers an adequate explanation.

For one thing, belief in God remains high amongst Catholics. Virtually all churchgoing Catholics believe in God, as do 70 % of self-identified Catholics taken as a whole.^3^ Catholics also report a rather high level of personal spiritual practice. Moreover, when asked about their activities over the last month, over 40 % say that they have prayed, a fifth that they have visited places which feel sacred or holy, the same number that they have taken regular time to be alone and still the mind, and 8 % that they have meditated. More than one in ten adherent Catholics read sacred and spiritual writings on a monthly basis, and the same number report “feeling a deep connection with nature/the earth.”

So most Catholics have not simply been secularising – abandoning faith in something “beyond”, to use Berger’s useful definition of religion – altogether. What they seem to have fallen out of love with is “religion” and their Church. As Clive Field (2014) has argued with regard to Britain, and Mark Chaves (1994) with regard to the USA, this is a crisis of religious authority more than one of religion. My surveys find that a full 96 % of self-identified Catholics say they take no authority *at all* from their religious leaders, local or otherwise. They may respect or admire them, but they don’t do what they say. Similarly, the practice of churchgoing has fallen away amongst younger Catholics: 1 in 3 over-60s attend weekly, but only 1 in 8 of those under 60. And only 36 % of Catholics say that they view the Catholic Church as a positive force in society.^4^

In other words, a growing proportion of Catholics, especially younger generations, retain the Catholic label and some elements of religiosity, but have distanced themselves from Catholicism as an authoritative “package”, and an off-the-shelf religious identity.

## “Non-religious” Britain

Standing back to look at Britain as a whole, it is clear that what we find amongst Catholics is not atypical. Neither the categories of denominational and religious identification (e.g., Christian, Hindu, Catholic), nor those of religious or secular, signify in the way they once did. A greater pluralism seems to have overwhelmed them, and rendered them much less useful than they once were.

The single most important, indeed epoch-making, fact about religion in Britain today is that the proportion of people identifying as having “no religion” is set to equal or overtake that of “Christians” within a few decades. Indeed, as Table 1 shows, if religions other than Christianity are taken out of the picture, “none” has already become the majority identity for British people under 50. According to my surveys, 38 % of the population now report having “no religion”, and the proportion grows to nearly half of young adults aged under 30, of whom only about a quarter identify with any Christian denomination. 5 Amongst those over 60 the situation is more than reversed, with 27 % having “no religion” and 58 % a Christian affiliation.^6^

**Table 1:** religion and no-religion by age

Age cohort	No religion	Christian	Other religion (including those who prefer not to state their religion)	No religion as % of the population (excluding Other)
18–19	55 %	20 %	25 %	73 %
20s	47 %	26 %	27 %	65 %
30s	44 %	28 %	28 %	61 %
40s	41 %	40 %	19 %	51 %
50s	36 %	47 %	17 %	43 %
60s	29 %	56 %	15 %	34 %
70s	21 %	62 %	17 %	26 %
80s	14 %	64 %	22 %	17 %
Total	38 %	41 %	21 %	48 %

However, a really crucial point is that “no-religion” does not simply equate to “secular” and “atheist”. Whilst only 16 % of nones believe in God, most are indifferent about the issue of God’s existence, rather than certain about “His” non-existence. Similarly, just 7 % of the population say they are influenced by humanism or secularism, and, as Table 2 shows, when people are given an additional option besides the conventional “religious, spiritual, both, none”, a plurality opt to take it. The idea that the British are uniformly drifting towards a thoroughgoing atheism resembling that of Richard Dawkins, is false.

**Table 2:** Spiritual, religious, other

Which, if any, of the following best describes you?	Nones	All
A spiritual person	12 %	15 %
A religious person	1 %	8 %
Both spiritual and religious	1 %	10 %
I would not describe myself, or my values and beliefs, as spiritual or religious	67 %	48 %
None of these	17 %	13 %
Dont know	3 %	6 %

Only a small proportion of those who report having no religion are actively hostile to religion. I identified such “hostile nones” as those who say they have no religion, are atheist, and agree that both the Church of England and the Roman Catholic Church are each a negative force in society. They amount to just 13 % of nones, and 5 % of the population. These “secularists” are disproportionately male, and are just as likely to be found amongst older as younger generations. They do not appear to be growing anything like as fast as the unaffiliated “nones”, and there are no more of them in younger than older generations.

Moroever, indifference to “religion” is not unique to “nones.” It is shared by a significant number of people who identify as Jewish, Christian, Buddhist etc. In total, only 47 % of those who report a religious affiliation describe themselves as religious and/or spiritual, and over a quarter say they are not influenced by religion. Equally, there are sizeable numbers of religiously-affiliated people who are atheists and agnostics – 63 % of those who identify as Buddhist, 42 % of those who state their religion as Jewish, 33 % of all Christians, 11 % of Muslims.^7^

## All Shook Up

Clearly old categories for understanding pluralism don’t seem to be working very well. Most people can’t easily be categorized as Christian, Buddhist, Jewish or whatever. Even if they tick that box on a survey, they defy the norms of what Christians (or Catholics), Jews, or Buddhists are meant to be like. They may believe in God, but say they are not religious; they may say they are Catholic but not believe in God; they may say they are Jewish but not influenced by religion – and so on.

Berger (2014) has a good explanation for this messiness. He reminds us that people are not consistent. They are different in different social contexts, and modern people live in highly differentiated societies. So someone may be very secular when she is at work as a surgeon performing an operation, but very religious when she is at home lighting Shabbat candles. As Nancy Ammerman (2014) points out in a response, however, many people in the USA these days seem to be complexly, pluralistically, religious and secular even when they are within a single institutional domain. So the surgeon may say a prayer when she gets to a tricky part of an operation, and may be thinking about something decidedly secular when she lights the candles. Qualitative research shows this better than qualitative. And the evidence is mounting up. People don’t fit the categories which the state, religious leaders, and many academics continue to use. Ammerman points out that a lot of the stories they tell about themselves aren’t clearly religious or secular either – the categories just don’t fit very well.

My surveys point to the same blurring of boundaries. More and more British as well as US people are “non-religious”, but this does not mean they are secular. “Non-religion” is not really an identity category at all, it’s an artefact of pre-existing, modern survey categories – a pollster’s clumsy recognition that the standard categories of Christian, Muslim, Buddhist have ceased to be relevant to an increasing number of people. And even those who can still tick the old religious boxes don’t necessarily behave or believe in the way they are expected to. The categories of “nones” and “non-religion” are chiefly important as a sign that the old classifications are breaking down. My suggestion is that we are seeing the arrival of a third, intensified, kind of pluralism: a pluralism of religious de-differentiation and deregulation.

## The Pluralism of De-differentiation

This third kind of religious pluralism is evident at the level of individual lives, and has a social-structural correlate in the de-differentiation of functions and sectors of post-industrial societies. Functional differentiation is, of course, a particular historical episode, associated with industrial modernity and the modern secular state. It is under pressure from under various late modern pressures, including the realisation that the ‘wicked problems’ facing the world today – from climate change to obesity – cross many different social domains, and require connected responses. This situation has important implications for religious pluralism.

Social differentiation did not just undermine religion, reducing its public sway, it also changed it. As Casanova (1994) argued so influentially, the most reliable insight of modern secularisation theories was that the differentiation of western societies denuded religion of its previous extension across society and stripped away many of its roles. It consolidated a more specialised and purely “religious” sector, with a discrete set of activities, and clear boundaries. This process was reinforced by the growth of modern colonial and colonial-national secular states. It was cemented by religious elites themselves, who evolved into more professionalised religious functionaries working within bureaucratic forms of religious structure, with clear roles and rewards and, often, state-recognition. In Britain this was all played out in relation to the historic religions and denominations, such as the Roman Catholic, Anglican, ‘Free’ Protestant, and Jewish. As clergy took more power to themselves, the role of laity (often female) in both governance and voluntary activity diminished accordingly (Brown and Woodhead 2016).

Both nationally and globally, this process tended to reify the existence and power of a small, authorised set of “world religions”, and denominations within them. They were characterised by some combination of a male leadership and clear teachings, practices and boundaries of orthodoxy or orthopraxy. State bureaucracy encouraged this process, which made classification, representation, enumeration, communication and control easier. There is a colonial and then a post-colonial phase to this process.

Recent intensified globalisation both strengthens and weakens such religious reification. It strengthens because religions respond by strengthening internal transnational links, sharpening their global ‘brand’, consolidating themselves in relation to transnational political and other arrangements, and defining themselves in relation to one another (Beyer 2006). But there are also powerful countervailing, ‘de-differentiating’ forces at work in late modernity.

One of the most important acids of the modern religious settlement is growing affluence worldwide, the creation of a global middle class, and expanding individual expectation of “voice and choice”. The latter, supported by both democracy and consumer capitalism, is starkest in relation to women’s often dramatically improved status and power, but evident more widely. At the same time, there is a massive expansion of education and a startling deregulation of information, both aided by the internet and the digital revolution. This undermines the virtual monopoly of religious knowledge and practice which religious professionals were able to consolidate in the modern era. Religious ideas and symbols float free on a scale never seen before, becoming available to any “seeker”, whilst social-networking media make it much easier to establish new religious networks and groupings whether real, virtual or both. These interlinked processes undermine the established entitlements of state-recognised religious leaders, and the bureaucratic and bounded forms of religious organisation on which their power rests become something of a liability as flexibility and meaningful lay participation become more essential to success.

So on the one hand, “modern” religious authorities lose their ability to police their own boundaries and maintain control and purity, whilst on the other hand, ordinary people feel new entitlements in relation to religion, as in other areas of their lives, and wish to think and choose for themselves rather than merely obey and be dutiful. In Britain, for example, my surveys of views on a range of ethical issues reveal that the overwhelming majority of the population (over 90 %) are liberal in terms of their commitment to individual choice rather than some other authority, and that most say that they rely on their own judgement and conscience to make important decisions (whether or not this is true in reality, this is their favoured perception). Not surprisingly, many such liberals are critical of what they see as the authoritarianism of established forms of religion. Some abandon it altogether, a few become actively hostile, others actively seek, find and create spiritual alternatives. But most draw on select elements of religion which they still find meaningful (e.g., life-course rituals, various symbols and narratives), abandon other elements (e.g., membership and regular churchgoing), and meld them all together with a wide range of other sources of significance.

This is what I mean by the new, intensified form of de-differentiated religious pluralism. It accounts for the fact that such a large and growing number of people now think of themselves as having “no religion”, for by this they mean the “packaged”, “dogmatic” religions of modern societies. They are rejecting something very particular, not necessarily the much broader phenonomenon of religion, or all aspcts of it, or ways it may be recreated. They do not necessarily become atheists, or abandon the belief that there are things beyond this life which give it meaning. Most do not abandon all aspects of their inherited religions to become neatly and hygienically secular, and they are not usually neatly religious in some domains of their life and secular in others. Nor do they resort to some sort of untrammelled “pick and mix” approach to religion: they are limited in what they “pick” by the religions with which they have had contact, and by the normal constraints of ethnicity, class, education, and the knowledge and networks to which they give access.

Importantly, people are decreasingly likely to think or act in terms of a neatly differentiated religious realm – such as that so well signified during the modern period by churchgoing on a Sunday morning. They are increasingly likely to engage with spiritual meaning across the various compartments of their life, and not merely in private. Consider, for example, how mindfulness meditation is now practised in healthcare settings, workplaces and so on, or in which spiritual and moral development is integral schooling in Britain.

Far from being corrosive of religion per se, this de-differentiating and increasingly de-regulated situation provides enormous opportunities for the new – and old – religious providers and entrepreneurs who are able to take advantage of it. Bewildering numbers set up stall in the new religious market place, offering an enormous variety of “products” for various niches. Many still draw on the existing “world” religious traditions, indeed some claim to be their uniquely faithful representatives – religious fundamentalists being a good example. Others at the opposite end of the spectrum rework or self-consciously invent new religions for a post-modern clientele (Cusack 2010). The entire charismatic upsurge, and much of what is happening to Islam, can be helpfully understood in this simultaneously differentiated and de-differentiating context, in which market forces become as important as state ones. Charismatic forms of authority find new opportunities as traditional and bureaucratic forms of leadership, both religious and secular, struggle to command respect. The forms of religion which are growing fastest in the world have in common the fact that they are fantastically disorganised, allow considerable lay participation, blur the lay-clerical boundary, are entrepreneurial, experiment with new, often charismatic, forms of authority, and are often rather institutionally-fragile and short-lived.

In this context, the old religions of modernity live on, desperately trying to shore up their defences and find new opportunities. Many try to sharpen their boundaries with clear statements of belief, strictness in moral teaching, and so on. Having a charismatic leader, like Pope Francis, may help, but will not guarantee obedience or faithful allegiance. Nevertheless, such religion continues to be supported by the fact that states, and international governing alliances, still recognise, register and otherwise legitimate these old forms of religion and their leaders (as do many voluntary organisations, as well as civil-society initiaitves like “inter-faith dialogue”). So at the macro-level in particular the differentiated, modern, religious order continues, even though at the meso- and micro-level it is increasingly hollowed-out and “polluted”.

I believe that many late-modern societies, including Britain and the USA, are thus in a transitional phase at the moment, in which the differentiated modern mode of religion persists alongside new de-differentiated, proliferating alternatives. Because the former is much more threatened by the latter than vice versa, one of its main defensive strategies is to deny that new forms of religion count as religion at all. Some academics take the same approach. But those involved in the new forms of “religion” are generally untroubled by having the label denied them. The growing association of religion with violence, reinforced by Islamic terrorism, only strengthens their desire to distance themselves from the toxic brand which is religion. A few prefer the word “spirituality”, but a growing number simply identify as having “no religion”, without necessarily losing interest or involvement in many aspects of what religion can provide.

The fact that we are in transition is revealed by the generational split in religious identification. The old categories of religious belonging remain relevant to those aged 60 and above (a majority of whom are Christian in Britain), but are increasingly less applicable to younger cohorts. Even those who still accept these labels, particularly in the middle age cohorts, fill them with a much more mixed content than religious and political leaders would like. Meanwhile, the old-style modern religions, most notably the churches, often become increasingly clerical and authoritarian. It is a mutually-reinforcing dynamic: the top-down tendency to purify fights against countervailing trends of de-differentiation and mixing from bottom-up.

None of this is completely new. Historians have taught us that even in medieval Catholic Europe ordinary people might hold or develop idiosyncratic and far-from-orthodox versions of religion. Some religions, like Hinduism, were always more resistant to systematisation and rationalization. What late, globalized, modernity does is to make the process of democratized reinvention and plundering much easier and less costly by on the one hand unlocking religious resources, and on the other hand undermining the power of traditional religious authorities to control them. This greatly enhances the capacity of ordinary believers to “do religion” for themselves, and a culture which privileges individual voice and choice only makes this seem more desirable.

## The Blurring of the Religious and Secular

Berger’s paradigm of twin pluralisms offers an extremely useful tool for making sense of religion in the modern world. The lens of religious pluralism helps us see how different religious traditions co-exist and relate within a given society, and what this means for their plausibility. It becomes more powerful when taken together with the lens of religious-secular pluralism. This not only helps us differentiate between various national state regimes in terms of their self-understandings, protocols, and modes of governance of religion, it also helps us look at different sectors of society – e.g., schools, hospitals – their self-representations, relations with religion, and so on. Both forms of pluralism are bound up with modern functionally differentiated societies, and with the modern nation states so central to them. They have been intensified by the flows of globalization, and its compression of space and time. Using both lenses, we understand much more about how individual lives are affected by pluralism.

Increasingly, this twin pluralism is joined by a new form of pluralism characteristic of post-industrial, affluent, highly-educated, late modern societies. It is bound up not with differentiation but with de-differentiation, and is a pluralism which has to do not just with clearly demarcated social functions and domains and their religious or secular profiles, but the breakdown of clear boundaries between them. Religion has leaked into areas of life from which it was temporarily exiled by modern projects; the religious-secular distinction becomes blurred or meaningless; the category of religion becomes toxic; the reality of religion as a separate domain of socio-political life wanes; religious professionals lose status and authority; people become their own priest. Increasing numbers of us inhabit the complex intersections between these three kinds of pluralism, as the old religious order is overtaken by something new.

## Footnotes

1 Two population surveys were carried out in January and June 2013. They are representative of adults aged 18+ in Great Britain excluding Northern Ireland. They were supplemented by a third survey completed by a nationally-representative sample of 1062 Catholics. The research was funded by two of the UK’s research councils, designed by Woodhead, and administered by YouGov. Data available at http://faithdebates.org.uk/research/.

2 My surveys can distinguish between different kinds of Catholics, including churchgoers and non-churchgoers. In what follows I am referring to *all* self-identified Catholics, unless otherwise indicated. Churchgoers are not as distinct from their non-churchgoing brethren as might be imagined. See Woodhead (2013a, b).

3 As Berger would predict, however, they believe with less certainty: a third of Catholics in their 20s say there is “definitely” a God, compared with 57 % of over-60s.

4 When those who take an actively negative view of the Church are asked their reasons, the most popular are: that the Church discriminates against women and gay people, because of the child abuse scandals, because it is hypocritical, and because it is too morally conservative.

5 The 2012 British Social Attitudes Survey (*n =* 3248) reports 48 % saying “no religion”, and the 2011 Census for England and Wales 25 %. The variation may be partly explained by the fact the Census may be filled in by the head of household on behalf of children and by the form of the question. Fewer people seem to opt for “no religion” when given the option of “Christian”, as on the Census. More report “no religion” when the option is “CofE”, “Catholic”, “Baptist” etc., as on the BSA survey and my YouGov surveys.

6 the balance of people either do not wish to state, or state their religion as “other” or a non-Christian faith.

7 “Nones” are more liberal than the rest of the population in their attitudes to personal morality, and there is a clear age effect with young people being in general more liberal. For example, among those under 50, nones believe that gay marriage is right by a margin of 59 %; the rest of the population is also in favour, but by the much narrower margin of 10 %.
